# Ethnicity-Specific Features of COVID-19 Among Arabs, Africans, South Asians, East Asians, and Caucasians in the United Arab Emirates

**DOI:** 10.3389/fcimb.2021.773141

**Published:** 2022-03-16

**Authors:** Fatmah Al Zahmi, Tetiana Habuza, Rasha Awawdeh, Hossam Elshekhali, Martin Lee, Nassim Salamin, Ruhina Sajid, Dhanya Kiran, Sanjay Nihalani, Darya Smetanina, Tatsiana Talako, Klaus Neidl-Van Gorkom, Nazar Zaki, Tom Loney, Yauhen Statsenko

**Affiliations:** ^1^ Mediclinic Parkview Hospital, Dubai, United Arab Emirates; ^2^ College of Medicine, Mohammed Bin Rashid University of Medicine and Health Sciences, Dubai, United Arab Emirates; ^3^ College of Information Technology, United Arab Emirates University, Al Ain, United Arab Emirates; ^4^ Big Data Analytics Center, United Arab Emirates University, Al Ain, United Arab Emirates; ^5^ College of Medicine and Health Sciences, United Arab Emirates University, Al Ain, United Arab Emirates; ^6^ Belarusian Medical Academy of Postgraduate Education, Minsk, Belarus; ^7^ Minsk Scientific and Practical Center for Surgery, Transplantology and Hematology, Minsk, Belarus

**Keywords:** COVID-19, ethnicity, Middle East, Gulf region, UAE, machine learning, viral pneumonia, host organism

## Abstract

**Background:**

Dubai (United Arab Emirates; UAE) has a multi-national population which makes it exceptionally interesting study sample because of its unique demographic factors.

**Objective:**

To stratify the risk factors for the multinational society of the UAE.

**Methods:**

A retrospective chart review of 560 patients sequentially admitted to inpatient care with laboratory confirmed COVID-19 was conducted. We studied patients’ demographics, clinical features, laboratory results, disease severity, and outcomes. The parameters were compared across different ethnic groups using tree-based estimators to rank the ethnicity-specific disease features. We trained ML classification algorithms to build a model of ethnic specificity of COVID-19 based on clinical presentation and laboratory findings on admission.

**Results:**

Out of 560 patients, 43.6% were South Asians, 26.4% Middle Easterns, 16.8% East Asians, 10.7% Caucasians, and 2.5% are under others. UAE nationals represented half of the Middle Eastern patients, and 13% of the entire cohort. Hypertension was the most common comorbidity in COVID-19 patients. Subjective complaint of fever and cough were the chief presenting symptoms. Two-thirds of the patients had either a mild disease or were asymptomatic. Only 20% of the entire cohort needed oxygen therapy, and 12% needed ICU admission. Forty patients (~7%) needed invasive ventilation and fifteen patients died (2.7%). We observed differences in disease severity among different ethnic groups. Caucasian or East-Asian COVID-19 patients tended to have a more severe disease despite a lower risk profile. In contrast to this, Middle Eastern COVID-19 patients had a higher risk factor profile, but they did not differ markedly in disease severity from the other ethnic groups. There was no noticeable difference between the Middle Eastern subethnicities—Arabs and Africans—in disease severity (p = 0.81). However, there were disparities in the SOFA score, D-dimer (p = 0.015), fibrinogen (p = 0.007), and background diseases (hypertension, p = 0.003; diabetes and smoking, p = 0.045) between the subethnicities.

**Conclusion:**

We observed variations in disease severity among different ethnic groups. The high accuracy (average AUC = 0.9586) of the ethnicity classification model based on the laboratory and clinical findings suggests the presence of ethnic-specific disease features. Larger studies are needed to explore the role of ethnicity in COVID-19 disease features.

## 1 Introduction

During the pandemic, the impact of coronavirus disease 2019 (COVID-19) on society varied considerably from country to country. To compare different nations, researchers estimated the impact with case fatality ratio (CFR). In the middle of 2020, the CFR was 3.7% in mainland China, 15.1% in the UK, and 14.2% in Italy (Grasselli et al., 2020; Mortality Analysis, 2020; Yang et al., 2020). Many factors may account for the difference in case fatality ratio across world regions, e.g., population density and settlement, the proportion of the elderly in the society, the affordability and accessibility of national healthcare systems, and the *ethnic background*, which implies genetic variation.

Studies on ethnic disparities of COVID-19 are challenging since there is no well-defined concept of ethnicity. On the one hand, this term refers to self-identification of people with a particular cultural group based on customs, norms, and ideologies. On the other hand, ethnicity is the cultural and genetic heritage of the person’s ancestors. The country of birth may inappropriately identify ethnicity because of the global tendency towards migration (Clarke et al., 2008). Another valid biological category for medical studies is race. Hypothetically, there is an association between genes that determine race and health. This does not comply with the data that genetic variations within races are more pronounced than between them (Egede, 2006).

Several studies and systematic reviews tried to explore whether ethnicity was a risk factor for severe COVID-19 disease form. However, the relationship between ethnicity and severe acute respiratory syndrome-related coronavirus 2 (SARS-CoV-2) infection remains uncertain (Pan et al., 2020). There are some inconsistencies in the findings on the association between ethnicity and clinical outcomes including hospitalization (Sze et al., 2020). However, a common limitation of such studies is that they focus on the West European or North American communities and consider other ethnicities as minorities. Though important for clinical risk stratification and proper patient management, such data are limited for multinational communities of the Gulf region with only few papers providing information on this issue (Almazeedi et al., 2020; Hannawi et al., 2021).

There is ambiguity regarding factors that account for the dissimilarities in COVID-19. Some authors show importance of disparities in the amount and location of adipose tissue whereas other authors point out a role of socioeconomic disparity (e.g., food insecurity and involvement in high-risk frontline jobs) (Islam et al., 2020; Krams et al., 2020). Environmental factors can also contribute to the diversity in the course of SARS-CoV-2 infection among people of different origins. The community of Dubai Emirate (United Arab Emirates; UAE) is an exceptionally interesting study sample because of its unique set of environmental, population, and economic factors. There is a pressing need to investigate these aspects of COVID-19 because this will contribute to proper risk management and foster further development of the community medicine (Habuza et al., 2021).

### 1.1 Studies on Ethnicity-Related Dissimilarities of COVID-19 in the Middle East

Commonly, researchers from the Middle East compare ethnicity subgroups by such aspects as migration during the pandemic and mental state. They do not explore an association between COVID-19 severity and outcomes with the patient’s race or ethnicity. Some studies provide descriptive statistics on ethnicity and citizenship of the participants (Al-Rifai et al., 2021; Hannawi et al., 2021) including pediatric patients (Elghoudi et al., 2020; Al-Rifai et al., 2021). Few of them categorize patients into Arabs and Asians to analyze their racial difference in disease severity and outcomes with adjustment for comorbidities (Ali et al., 2021; Deeb et al., 2021). Unique research of antibody titers and epitope coverage is carried out predominantly on Caucasians and South Asians with few Middle Eastern patients included (Smith et al., 2021). One study in Israel compared the impact of COVID-19 on Jewish and Arab populations (Haklai et al., 2021).

### 1.2 Studies on Ethnic Features of COVID-19 Across the World

**Figure 1:**
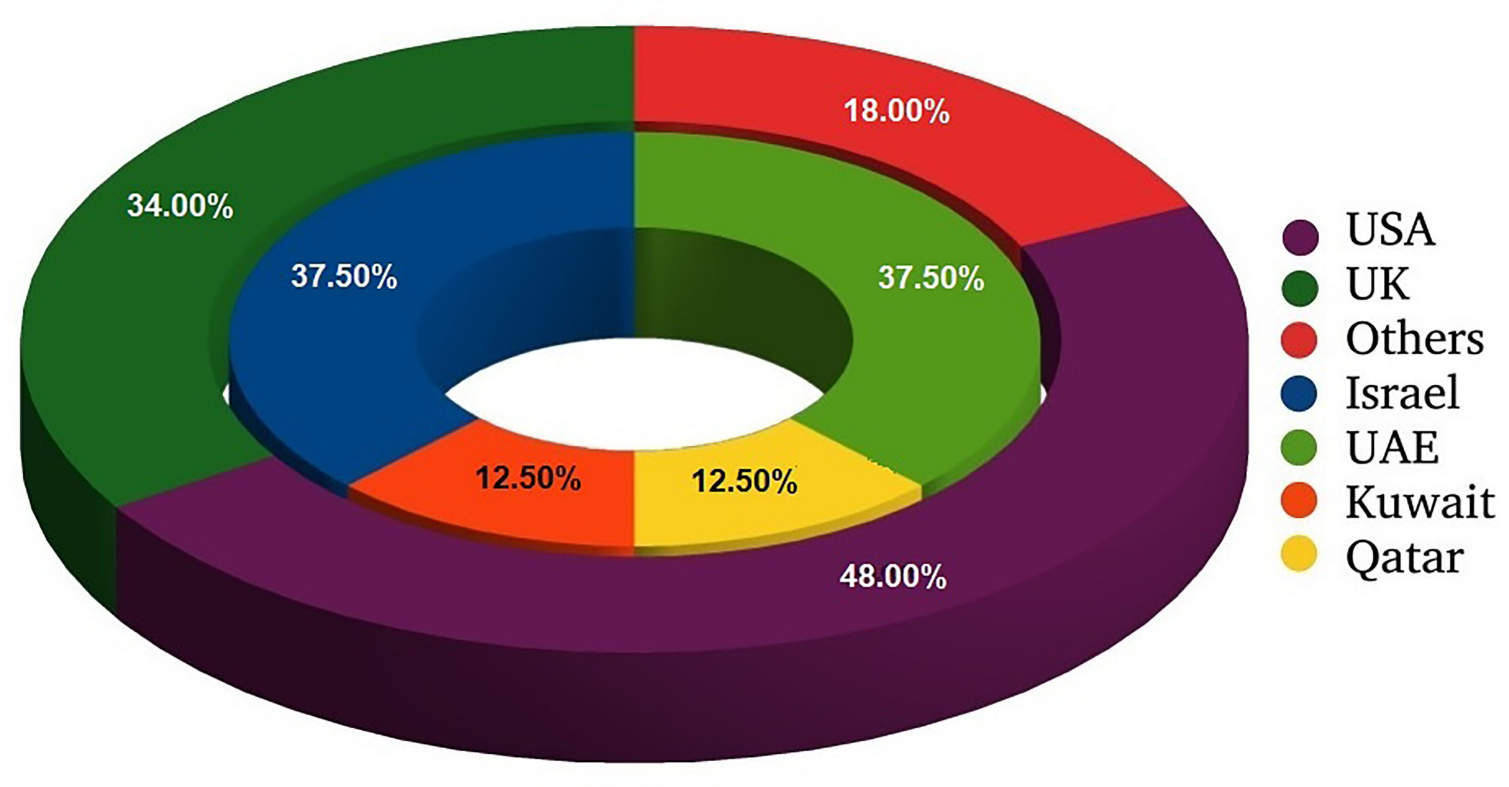
Distribution of research papers on ethnical issues of COVID-19 with regard to location of study. External circle presents all analyzed studies; internal one shows information on studies conducted in Middle East.

**Figure 2:**
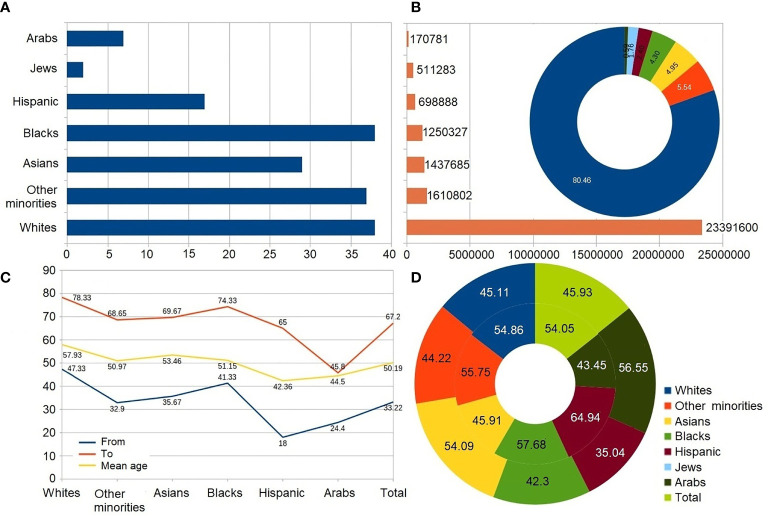
Analysis of the ethnicity-based research papers. Circle diagrams report data in percentages. Results are calculated as average value of data reported in papers. **(A)** Number of papers. **(B)** Size of study cohort; circle diagram represents percentage out of all studied population. **(C)** Average age of participants. **(D)** Gender distribution. External/internal circle represents male/female rate.

**Table 1** and both **Figures 1** and **2** summarize study cohorts of 50 open-access papers that reported original findings on the dissimilarities of COVID-19 among ethnic groups. The papers were retrieved consecutively from the Google Scholar search engine with the query comprising the following keywords: “race, ethnicity, COVID-19”. In this paper, we want to characterize research methodology traditionally used in such studies and discuss some of their limitations. The majority of the studies related to ethnicity and COVID-19 outcomes were conducted by scientists from either the USA or the UK (Bassett et al., 2020; Hsu et al., 2020; Karaca-Mandic et al., 2021; Lundon et al., 2020; Rentsch et al., 2020; Gianfrancesco et al., 2021; Kabarriti et al., 2020). The US population is traditionally divided into Hispanic, non-Hispanic Whites, non-Hispanic Blacks, Asians, and certain minorities including Alaska Natives. The above-mentioned studies did not include Arabs as a separate ethnic entity. Many studies have been devoted to an association between mortality from COVID-19 and race (Bassett et al., 2020; Hsu et al., 2020; Kabarriti et al., 2020; Lundon et al., 2020; Gold et al., 2020; Golestaneh et al., 2020; Gianfrancesco et al., 2021; Price-Haywood et al., 2020; Rentsch et al., 2020; Rossen et al., 2020), an effect of ethnicity on *hospitalization* (Hsu et al., 2020; Lundon et al., 2020; Price-Haywood et al., 2020; Gianfrancesco et al., 2021), *admission to intensive care unit* (ICU) (Hsu et al., 2020), and *the need for mechanical lung ventilation* (Hsu et al., 2020; Lundon et al., 2020). Few studies explain the influence of ethnicity on *COVID-19 outcomes* in a socio-demographic context (Lundon et al., 2020; Raifman and Raifman, 2020; Millett et al., 2020). Some scientists from North America compared the mortality rate among people of different origin before and after the COVID-19 pandemic (Golestaneh et al., 2020). Authors compared laboratory findings among ethnic subgroups of patients with COVID-19 (Price-Haywood et al., 2020). Researchers adjust the study subgroups with regard to comorbidities and background conditions (Lundon et al., 2020; Golestaneh et al., 2020; Gianfrancesco et al., 2021).

**Table 1:** Exploratory analysis on number, age, and gender of participants in ethnic studies.

Ethnicity	Papers	Total	M, %	F, %	Age			
					From	To	Mean	Std
Whites	38	23,391,600	45.11	54.86	47.33	78.33	57.93	11.66
Other Minorities	37	1,610,802	44.22	55.75	32.90	68.65	50.97	13.09
Asians	29	1,437,685	54.09	45.91	35.67	69.67	53.46	6.46
Blacks	38	1,250,327	42.30	57.68	41.33	74.33	51.15	9.86
Hispanic	17	698,888	35.04	64.94	18.00	65.00	42.36	10.37
Jews	2	511,283						
Arabs	7	170,781	56.55	43.45	24.40	45.80	44.50	
Total	50	29,071,366	45.93	54.05	33.22	67.20	50.19	9.22

For epidemiological analysis, scientists from the UK aggregate data from the UK Biobank (Niedzwiedz et al., 2020), National Healthcare Service registry (Aldridge et al., 2020), Open SAFELY platform (Collaborative, 2021), and a few hospitals (Sapey et al., 2020; Apea et al., 2021). The specialists compare Blacks, Whites, South and East Asians, and minority ethnic groups, and they do not study peculiar features of COVID-19 among Arabs as a separate ethnic group (Alaa et al., 2020; Aldridge et al., 2020; Harrison et al., 2020; Hull et al., 2020; Niedzwiedz et al., 2020; Patel et al., 2020; Sapey et al., 2020; Apea et al., 2021; Collaborative, 2021; Nafilyan et al., 2021). The research objectives of the present study are as follows: investigate the relationship between race and incidence of SARS- CoV-2 infection (Niedzwiedz et al., 2020; Collaborative, 2021), disease *outcomes* (Harrison et al., 2020; Patel et al., 2020; Alaa et al., 2020), *and both hospitalization and ICU admission* (Apea et al., 2021). To adjust the supposed risk factors, various authors stratify the study samples by age, body mass index (BMI), comorbidities (Harrison et al., 2020), the number of people in the household, the accommodation type, income, and other factors (Nafilyan et al., 2021). While the majority of researchers aggregate data on epidemiology and patient management, few of them integrate ethnic-specific patterns of COVID-19 with laboratory findings, e.g., biochemical data of positively tested individuals (Apea et al., 2021).

### 1.3 The Timeline of the Spread of COVID-19 in the UAE

The United Arab Emirates was among the first countries to implement strict measures to control the pandemic, e.g., closing the national borders, limiting internal public movements and gatherings, shutting down schools, and the use of distant learning, and implementing work from home protocols. These measures smoothed the peak of the disease incidence and allowed the healthcare system to re-organize hospitals to effectively manage the case load and the COVID-19 outbreak, creating multiple field hospitals that can handle mild cases and converting several hotels to isolation facilities run by healthcare staff.

**Figure 3:**
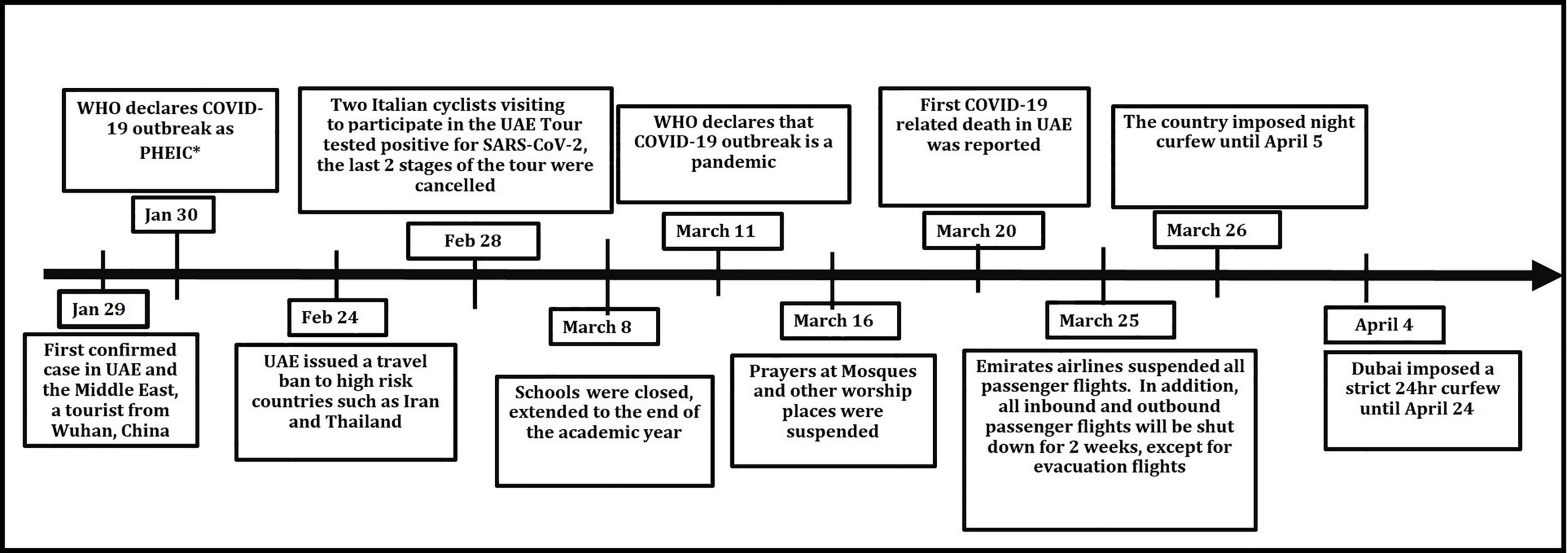
Timeline of COVID-19 outbreak in UAE.

On January 29 2020, the UAE announced its first confirmed case of COVID-19. It was the first country in the Middle East to register a case of COVID-19. The first patient was a Chinese tourist who arrived in the UAE from Wuhan on January 16. By the end of January, there had been five confirmed cases of COVID-19 in the UAE. On January 30, WHO declared the novel coronavirus outbreak a PHEIC (Public Health Emergency of International Concern) (Tayoun et al., 2020). **Figure 3** shows the major steps taken by the UAE government to limit the spread of COVID-19.

The UAE population has increased substantially in the last 2 decades as a result of the remarkable growth in its economy. The economic growth has led to the influx of expatriate workers from all over the world. This accounts for the uneven distribution of age (more than two-thirds of the population is between 25 and 45 years of age) and the heterogeneous ethnic backgrounds. According to Dubai Statistics Center for 2019, the Dubai population was 3.3 million individuals, which constitutes 34% of the whole UAE population. Only 1.2% residents are older than 65 years of age (Wang et al., 2020; Yang et al., 2020; Zhou et al., 2020). Males represent 69.5% of the population. The vast majority of the individuals (92%) are non-UAE nationals, with 59.4% from South Asia and approximately 6% from the Philippines, which has led to a marked variation in sociodemographic structure of the UAE population.

## 2 Objectives

We intended to stratify the risk factors for the multinational society of Dubai. To address this objective, we had the following specific tasks:

- Explore the ethnic-specific features of COVID-19 by analyzing the disease course in people of different ethnic groups.
- Rank the most significant features that account for the ethnic-specific course of COVID-19 in the Dubai population.
- Build a model of ethnic specificity of COVID-19 based on the clinical presentation and laboratory findings on admission and evaluate its performance.

## 3 Materials and Methods

### 3.1 Study Sample

We obtained retrospective data routinely collected as part of standard primary and secondary care. The study sample comprised all COVID-19 patients consecutively admitted to Mediclinic Parkview Hospital, Dubai (UAE), from the date of the first confirmed case, February, 26, 2020, until May, 31, 2020. At the beginning of the pandemic, all the patients with COVID-19 verified by reverse-transcriptase polymerase chain reaction (PCR) were hospitalized to Mediclinic regardless of the disease severity or medical insurance coverage. This made our study cohort representative of the entire Dubai population. The cohort included many asymptomatic and mild cases. A thorough description of the flowchart for the management of COVID-19 patients is given in our recent paper (Statsenko et al., 2021). See the patient management with a flow chart in our recent paper (Statsenko et al., 2021). Details of the current study are provided below.

The inclusion criteria were as follows (1): aged 18 years old or above (2); positive SARS-CoV-2 real-time PCR from a nasopharyngeal swab; and (3) inpatient admission. Patients meeting the inclusion criteria were followed until discharge. The national guidelines regulated inpatient management. As a part of the standard of care, baseline blood tests and inflammatory markers were obtained. Multiplex PCR assays were used to test respiratory samples for influenza and other respiratory viruses. Supportive oxygen therapy was initiated if oxygen saturation measured with pulse oximeter dropped below 94% or respiratory rate increased above 30 breaths per minute. Patients who were clinically suspected of having superimposed bacterial pneumonia were administered empirical broad-spectrum antibiotics at the discretion of the treating physician. The antiviral and antimalarial therapies were guided by “National Guidelines for Clinical Management and Treatment of COVID-19”, a standardized guideline for all health sectors in the UAE (National Emergency Crisis and Disasters Management Authority, 2020).

It was a single-center study with a relatively short duration (3 months) as the UAE government standardized healthcare service for COVID-19 patients during this period in the following way. All the patients with a positive PCR test were hospitalized to either government-funded or private health facilities even if they were asymptomatic. The diagnostics and treatment of COVID-19 were provided free of charge in accordance with the National Guidelines (National Emergency Crisis and Disasters Management Authority, 2020). Because of this, the sample of the study is representative for the adult population of the country. Dubai Mediclinic is affiliated with the governmental medical school—Mohammed Bin Rashid University of Medicine and Health Sciences—and is the optimal center for medical teaching and research in Dubai.

### 3.2 Data Collection

We extracted the electronic health records of all consecutive patients with either admission or discharge diagnosis of COVID-19, coronavirus infection, unspecified, SARS-associated coronavirus as the cause of diseases classified elsewhere, other coronavirus as the cause of diseases classified elsewhere, and pneumonia due to SARS-associated coronavirus (ICD10 codes U07.1, B34.2, B97.21, B97.29, and J12.81). Patients with negative SARS-CoV-2 PCR were excluded from the study. The information from electronic health records was extracted using a standardized data collection form by trained researchers. The form was adapted from ISARIC Rapid Case Record Form (COVID-19 CASE RECORD, 2020), see *Subsection 3.3*.

Variables of interest were manually extracted from electronic health records. The abstraction team of 7 physicians was trained and supervised by the principal investigator. To ensure the accuracy of the data entered, the team worked in the following manner. A team member extracted the required information to a data collection sheet. Another physician double-checked the information from the data collection sheet before entering it to an Excel spreadsheet. Any discrepancies were resolved by the supervisor.

### 3.3 Dataset Description

In our study, we classified the subjects into the following ethnic groups: Middle Easterns (Arabs of both the UAE and non-UAE nationality, Africans from the countries mentioned in **Table 2**), South Asians (patients from Afghanistan, Bangladesh, Bhutan, Maldives, Nepal, India, Pakistan, and Sri Lanka), East Asians (patients from China, Hong Kong, Philippines, Taiwan, Japan, Mongolia, North Korea and South Korea), Caucasians, and others.

**Table 2:** Nationalities of Middle Eastern ethnic group.

Ethnicity	Subethnicity	Nationality	Number of cases
Middle Easterns 148 (100%)	Arabs 114 (77.03%)	UAE	73 (49.32%)
		Oman	8 (5.41%)
		Lebanon	5 (3.38%)
		Syrian	5 (3.38%)
		Jordon	5 (3.38%)
		Bahrain	3 (2.03%)
		Iran	3 (2.03%)
		Pakistan	2 (1.35%)
		Canadian Arab	2 (1.35%)
		Palestine	2 (1.35%)
		USA Arab	2 (1.35%)
		Yemen	1 (0.68%)
		Australian Arab	1 (0.68%)
		Iraq	1 (0.68%)
		French Arab	1 (0.68%)
	Africans 34 (22.97%)	Egypt	30 (20.27%)
		Sudan	3 (2.03%)
		Cameroon	1 (0.68%)

To determine the case severity, we used the UAE National Guideline for Clinical Management and Treatment of COVID-19 (National Emergency Crisis and Disasters Management Authority, 2020):

- *Asymptomatic form* refers to a patient with no symptoms.
- *Mild form*—clinical symptoms of upper respiratory tract infection and no signs of pneumonia.
- *Moderate form*—fever and respiratory symptoms with radiological findings of pneumonia.
- *Severe form*—any one of the following criteria: respiratory distress (respiratory rate > 30/min), oxygen saturation <93% at rest, P/F ratio of less than 300.
- *Critical form*—any of the following criteria: acute respiratory distress syndrome (ARDS) with P/F ratio < 200, sepsis, multi-organ failure, altered level of consciousness (GCS<13).

The list of variables comprising the complete cohort dataset is as follows:

- **Demographics features**. Patient’s age, gender, ethnicity, weight, height, body mass index (BMI), and occupation (for the patients who are healthcare or laboratory workers), travel history within 14 days prior to symptom onset, and exposure to a confirmed case of COVID-19.
- **Comorbidities**. History of chronic cardiac disease, hypertension, chronic lung disease, asthma, chronic kidney disease, diabetes, active malignant cancer, immunosuppressed state, human immunodeficiency virus infection (HIV), active smoking, and pregnancy status in female patients. Medication history: whether the patient was taking any of the following medications prior to the admission: angiotensin-converting enzyme inhibitors (ACE-I), angiotensin II receptor blockers (ARB), and/or non-steroidal anti-inflammatory drugs.
- **Symptoms at presentation**. Cough, sputum production, sore throat, chest pain, shortness of breath (SOB), fever, headache, confusion, nausea or vomiting, diarrhea, myalgia, malaise, and loss of smell or taste.
- **Vital signs**. Temperature, heart rate (HR BPM), respiratory rate, systolic (SBP) and diastolic blood pressure (DBP), SpO2, and sequential organ failure assessment (SOFA) score at the time of admission and at the time of transfer to ICU if applicable.
- **Laboratory findings**. The following parameters were collected *on admission and at the peak of illness*: white blood cell (WBC) count, lymphocyte count, platelet count, activated partial thromboplastin time (APTT), the activity of the enzymes—alanine aminotransferase (ALT), aspartate aminotransferase (AST), lactate dehydrogenase (LDH), creatine kinase (CK), and the concentration of the total bilirubin, D-dimer, creatinine, C-reactive protein (CRP), sodium ion, troponin, ferritin, and fibrinogen. Blood hemoglobin and serum sodium were recorded on admission.
- **Case management and clinical course**. Medications used: antiviral medication, azithromycin, other intravenous antibiotics, antimalarial, antifungal medication, IL-6 blocker “Tocilizumab”, convalescent plasma, steroids (either intravenous or oral), low-molecular-weight heparins, supplemental oxygen, invasive ventilation, vasopressors, and extracorporeal membrane oxygenation. The length of hospital stay, the duration between symptom onset and admission (in days), the duration between the first positive SARS-CoV-2 PCR and the first negative set (first of 2 consecutive negative PCRs), need for ICU care, and the duration of stay in ICU.
- **Complications**.
- *Septic shock* was defined according to the 2016 Third International Consensus Definition for Sepsis and Septic Shock (Singer et al., 2016).
- *Bacterial pneumonia* was diagnosed when patients showed clinical symptoms or signs of pneumonia and a positive culture of a new pathogen was obtained from lower respiratory tract specimens (qualified sputum, endotracheal aspirate, or bronchoalveolar lavage fluid) after admission.
- *Bacteremia* was diagnosed when patients showed clinical symptoms or signs of systemic infection and one or more positive blood culture that was not thought to be a contaminant.
- *ARDS* was diagnosed according to the Berlin definition (Force et al., 2012).
- *Acute cardiac injury* was diagnosed if serum levels of cardiac biomarkers (troponin I) were above the 99th percentile upper reference limit, or if new abnormalities were shown in echocardiography.
- *Acute kidney injury* was defined according to Kidney Disease Improving Global Outcomes (KDIGO) (Khwaja, 2012). It is based on the highest serum creatinine level and urine output. Specifically, the diagnosis could be made if there is an increase in serum creatinine levels by 0.3 mg/dl or greater (26.5 µmol/L or greater) within 48 h.
- *Liver injury* was diagnosed if there was an increase in liver enzymes (AST or ALT) over 3 times the upper limit of normal.
- *Seizure, meningitis*, or *encephalitis* confirmed by CSF analysis and culture, cardiac arrhythmia, cardiac arrest, myocarditis (if clearly documented by a cardiologist or an intensivist), new onset cardiomyopathy (if the baseline cardiac function is unknown, assume new), critical illness myopathy or neuropathy (if documented by an attending physician or diagnosed with the electrophysiologic testing), bleeding or disseminated intravascular coagulation (DIC), the use of renal replacement therapy, the development of pressure ulcer, and other complications.
- **Primary Outcome**. Discharged alive.

### 3.4 Statistical Analysis

The data were checked for accuracy and then for normality using the Shapiro–Wilk test; none of the attributes were normally distributed; the non-parametric tests were used to compare each pair of independent samples. The bivariate relationships between the features were assessed with the Mann–Whitney *U* test or Kruskal–Wallis test for the continuous variables, and with Fisher’s Exact test or Chi-square test for the quantitative ones. As we intended to find features inherent to the specific ethnic group, we also evaluated the differences between each group versus the others.

**Machine learning (ML) classification model**. We utilized ML algorithms to check if there were unique patterns within the data that can unambiguously identify the ethnic group (Middle Eastern, South Asian, East Asian, Caucasians, and Others). In our dataset, the ethnic group “Others” was in the minority (14 patients, 2.5%), so we excluded it from the analysis.

The list of variables used to build the model was as follows:

- *Physical examination on admission*: temperature, HR BPM, SBP, DBP, the time elapsed between two successive R-waves on the electrocardiogram (RR/min), oxygen saturation (SpO_2_), SpO_2_ on room air *vs*. oxygen therapy, Glasgow coma scale (GCS), and SOFA score.
- *Symptoms on admission*: cough, sputum, sore throat, chest pain, SOB, fever, headache, confusion, having any gastrointestinal symptom (e.g., nausea, vomiting, diarrhea), myalgia, malaise, and loss of smell or taste.
- *Laboratory findings on admission*: the count of platelets, WBC, and fractions of leukocytes; the concentration of hemoglobin, total bilirubin, D-dimer, creatinine, sodium, CRP, troponin, ferritin, and fibrinogen; the activity of ALT, AST, CK, and LDH; and the length of APTT.

**Feature selection**. To assess the importance of the features fed to the ML models as classifiers by ethnicity, we employed four ensemble tree-based estimators such as AdaBoost, Gradient Boosting, Random Forest, and Extra Trees. These models were trained on the whole dataset and used to rank the features in ascending order concerning their predictive potential.

## 4 Results

### 4.1 The Cross-Ethnic Groups

Out of 560 patients, 43.8% were South Asians, 26.4% were from the Middle East, 16.8% were East Asians, 10.7% were Caucasians, and 2.50% are under Others (see **Tables 2**, **3**). The UAE nationals represented half of the Middle Eastern patients, i.e., 13% of the entire cohort. Overall, males accounted for two-thirds of the study population, which remained true across different ethnic groups except for the East Asians where the gender distribution was almost equal. **Table 2** lists nationalities in the Middle Eastern ethnic group. The comparison of the patients of the Middle Eastern subethnicities—Arabs and Africans—is given in **Table 4**. There were marked differences in the SOFA score, the level of D-dimer (p = 0.015), and fibrinogen (p = 0.007) between the subethnic groups on admission. The pronounced disparity between the Arabs and Africans in the background diseases (hypertension - p = 0.003; diabetes and current smoking - p = 0.015) may account for the mentioned differences. We did not find a noticeable difference between the subethnic groups in disease severity (p = 0.81).

**Table 3:** Comparison of patients of different ethnic groups.

		Total	South Asians	Middle Easterns	East Asians	Caucasians	Others	*p*-value	Missing values
		*n* = 560	*n* = 244 (43.57%)	*n* = 148 (26.43%)	*n* = 94 (16.79%)	*n* = 60 (10.71%)	*n* = 14 (2.5%)		
**Patient age**		39.0 [33.0–49.0]	39.0 ± 11.35	38.5 ± 16.66	37.0 ± 8.86	43.5 ± 11.48*	38.5 ± 6.31	0.0933	
**Patient gender**	Female	189 (33.75%)	55 (22.54%)*	55 (37.16%)	50 (53.19%)*	22 (36.67%)	7 (50.0%)	**<0.0001**	
	Male	371 (66.25%)	189 (77.46%)*	93 (62.84%)	44 (46.81%)*	38 (63.33%)	7 (50.0%)		
**Comorbidities**									
Current smoking		36 (6.43%)	10 (4.1%)	15 (10.14%)*	4 (4.26%)	7 (11.67%)		**0.0401**	
Chronic cardiac disease		20 (3.57%)	8 (3.28%)	10 (6.76%)*	1 (1.06%)	1 (1.67%)		0.1214	
Hypertension		115 (20.54%)	47 (19.26%)	37 (25.0%)	21 (22.34%)	8 (13.33%)	2 (14.29%)	0.3449	
Asthma		38 (6.79%)	14 (5.74%)	9 (6.08%)	9 (9.57%)	6 (10.0%)		0.4489	
Chronic kidney disease		7 (1.25%)	2 (0.82%)	4 (2.7%)			1 (7.14%)		
Diabetes		98 (17.5%)	51 (20.9%)	37 (25.0%)*	8 (8.51%)*	1 (1.67%)*	1 (7.14%)	**<0.0001**	
Active malignant cancer		6 (1.07%)	2 (0.82%)	3 (2.03%)	1 (1.06%)				
**Physical examination**									
BMI	adm	27.0 [23.92–30.44]	26.5 ± 4.2	28.0 ± 7.03*	26.64 ± 5.37	26.85 ± 4.45	27.05 ± 3.72	0.2195	49.64%
Body temperature, °C	adm	37.0 [37.0–37.9]	37.0 ± 0.7	37.0 ± 0.62*	37.2 ± 0.74	37.0 ± 0.86	37.4 ± 0.71	**0.0343**	
HR BPM	adm	85.0 [78.0–95.0]	85.0 ± 14.08	85.0 ± 12.11	89.0 ± 13.68*	80.0 ± 14.97*	84.5 ± 12.19	**0.0014**	
SBP	adm	124.0 [114–135]	124.0 ± 16.02*	120.5 ± 16.12*	124.5 ± 20.31	123.5 ± 12.73	123.5 ± 14.63	0.0786	
DBP	adm	78.0 [70.0–84.0]	78.0 ± 10.98*	74.0 ± 9.69*	80.0 ± 11.46*	76.0 ± 9.88	72.0 ± 10.69	<0.0001	
RR/min	adm	18.0 [18.0–18.0]	18.0 ± 4.53*	18.0 ± 2.8*	18.0 ± 4.14	18.0 ± 1.48	18.0 ± 1.03	**0.0394**	
SOFA score	adm	0.0 [0.0–0.0]	0.0 ± 2.16	0.0 ± 1.09	0.0 ± 1.56	0.0 ± 0.7	0.0 ± 0.52	0.2733	0.71%
**Laboratory findings**									
WBC, ×10^9^/L	adm	5.8 [4.5–7.2]	6.1 ± 2.51*	5.2 ± 3.78*	5.95 ± 2.94	5.65 ± 4.55	4.2 ± 1.86*	**<0.0001**	0.54%
	min	5.5 [4.1–7.2]	6.0 ± 10.79*	4.7 ± 2.21*	6.0 ± 3.63	5.3 ± 5.48	3.6 ± 1.88*	**<0.0001**	0.54%
Platelets, ×10^9^/L	adm	224.0 [180–272]	227.0 ± 68.77	207.0 ± 72.21*	260.5 ± 106.96*	206.5 ± 55.27*	222.5 ± 57.78	**<0.0001**	0.36%
	min	224.0 [178.0–272.0]	224.0 ± 85.51	211.0 ± 77.6	260.0 ± 104.38*	207.0 ± 60.95*	222.5 ± 79.8	**<0.0001**	0.36%
Lymphocytes, ×10^9^/L	adm	1.55 [1.06–2.1]	1.62 ± 0.79	1.57 ± 0.71	1.52 ± 0.67	1.37 ± 0.75	1.04 ± 0.6	0.3291	0.54%
	min	1.49 [0.89–2.09]	1.54 ± 0.87	1.5 ± 0.76	1.43 ± 0.74	1.32 ± 3.91	1.04 ± 1.67	0.6228	0.54%
Neutrophils, ×10^9^/L	adm	3.43 [2.4–4.57]	3.72 ± 2.14*	2.94 ± 2.43*	3.56 ± 2.4	3.48 ± 2.93	2.24 ± 1.49	**5.7e-05**	0.54%
	min	3.24 [2.13–4.49]	3.55 ± 2.44*	2.57 ± 1.83*	3.6 ± 2.25*	2.89 ± 2.26	2.14 ± 1.5*	**3.3e-07**	0.54%
Neutropenia, <1×10^9^/L	adm	12 (2.14%)	2 (0.82%)	8 (5.41%)*	1 (1.06%)	1 (1.67%)		**0.0336**	0.54%
	min	20 (3.57%)	5 (2.05%)	11 (7.43%)*	1 (1.06%)	3 (5.0%)		**0.0309**	0.54%
NLR	adm	2.07 [1.31–3.49]	2.12 ± 4.39	1.89 ± 2.66*	2.48 ± 3.85	2.12 ± 3.17	1.9 ± 1.75	**0.0206**	0.54%
	min	2.06 [1.3–3.69]	2.14 ± 10.34*	1.78 ± 2.93*	2.56 ± 8.34*	1.82 ± 4.07	1.86 ± 2.16	**0.0013**	0.54%
LCR	adm	0.26 [0.05–0.96]	0.28 ± 17.31	0.25 ± 16.73	0.2 ± 12.6*	0.41 ± 3.04	0.33 ± 0.84	0.2146	10.89%
	peak	17.05 [1.61–61.85]	21.07 ± 80.43	15.79 ± 67.21	11.11 ± 57.17	18.64 ± 88.51	4.33 ± 692.68	0.6871	10.71%
T.bilirubin, μmol/L	adm	9.0 [6.0–12.6]	10.0 ± 6.87*	8.0 ± 5.06*	8.0 ± 5.77*	8.2 ± 4.58	7.0 ± 4.9	**<0.0001**	1.96%
	peak	9.85 [6.5–14.38]	10.4 ± 21.82*	8.6 ± 6.09*	9.5 ± 10.81	11.4 ± 8.1	9.0 ± 5.09	**<0.0001**	1.79%
ALT, U/L	adm	28.0 [17.25–47.75]	29.0 ± 33.34	25.0 ± 40.81	34.0 ± 38.83	25.0 ± 24.5	29.0 ± 23.13	0.1533	1.79%
	peak	32.0 [19.0–67.75]	35.0 ± 465.54	27.0 ± 5109.99*	37.0 ± 80.04	29.0 ± 58.7	29.0 ± 118.76	0.1017	1.79%
AST, U/L	adm	24.0 [18.0–36.22]	25.0 ± 25.33	23.0 ± 34.69*	28.0 ± 21.46	24.0 ± 16.12	24.5 ± 18.35	0.0709	1.79%
	peak	25.5 [19.0–44.0]	26.0 ± 447.91	25.0 ± 345.96*	29.0 ± 51.14*	24.5 ± 18.3	24.5 ± 65.43	0.1035	1.79%
D-dimer, mg/L	adm	0.4 [0.2–0.6]	0.3 ± 1.59	0.4 ± 0.51	0.4 ± 2.5	0.3 ± 0.44	0.4 ± 0.35	0.6844	15.36%
	peak	0.4 [0.3–0.7]	0.4 ± 4.65	0.4 ± 2.06	0.4 ± 4.15	0.35 ± 2.31	0.6 ± 0.34	0.4907	15.36%
APTT, s	adm	37.4 [35.0–41.0]	37.55 ± 10.97	37.0 ± 12.06	39.3 ± 4.96*	37.0 ± 4.96	35.5 ± 3.81	**<0.0373**	13.04%
	peak	38.0 [35.15–42.35]	38.0 ± 22.65	37.6 ± 13.07	40.8 ± 25.56*	37.0 ± 5.2	36.5 ± 5.04	**<0.0116**	13.04%
Creatinine, μmol/L	adm	76.1 [67.0–89.0]	79.8 ± 37.3*	75.3 ± 28.23	70.0 ± 31.39*	82.0 ± 17.44	68.5 ± 42.64	**0.0023**	1.07%
	peak	78.0 [67.78–91.0]	80.8 ± 57.55*	76.0 ± 31.03	70.0 ± 47.76*	83.5 ± 17.86	68.5 ± 42.64	**0.0009**	1.07%
CK, U/L	adm	106.0 [66.0–173.0]	119.5 ± 947.62*	84.0 ± 230.14*	117.0 ± 535.75	88.5 ± 102.47	105.5 ± 54.77	**<0.0005**	22.5%
	peak	109.5 [66–199]	127.0 ± 988.14*	86.0 ± 243.77*	122.5 ± 10275.55	88.5 ± 136.52	105.5 ± 96.62	**<0.0005**	22.32%
CRP, mg/L	adm	5.8 [1.75–27.0]	5.3 ± 64.11	5.9 ± 50.07	6.85 ± 68.44	5.35 ± 59.98	6.3 ± 43.76	0.8459	0.89%
	peak	6.5 [1.9–50.65]	5.9 ± 86.33	7.95 ± 64.16	8.3 ± 83.66	5.75 ± 67.84	6.3 ± 93.69	0.8856	0.89%
LDH, U/L	adm	192.0 [159.0–264.0]	195.0 ± 182.27*	181.0 ± 86.08*	235.0 ± 200.88*	175.0 ± 70.41	167.5 ± 123.58	**<0.0001**	16.96%
	peak	194.0 [160.0–280.0]	195.0 ± 626.18	183.0 ± 647.29*	238.0 ± 239.95*	175.0 ± 89.53	167.5 ± 125.35	**<0.0001**	16.96%
Troponin, ng/mL	adm	0.0 [0.0–0.0]	0.0 ± 0.83	0.0 ± 0.01	0.0 ± 0.01	0.0 ± 0.03	0.0 ± 0.0	0.5822	24.11%
	peak	0.0 [0.0–0.0]	0.0 ± 1.17*	0.0 ± 0.07	0.0 ± 0.1	0.0 ± 0.03	0.0 ± 0.0	0.2089	24.11%
Ferritin, ng/mL	adm	216.7 [84.5–475.5]	216.7 ± 1499.98	191.9 ± 535.61*	286.0 ± 1604.47	261.0 ± 524.67	289.0 ± 1231.74	0.0743	9.46%
	peak	230.0 [89.95–595.5]	230.0 ± 5730.89	205.0 ± 2834.62*	469.0 ± 3214.62	261.0 ± 590.38	321.6 ± 1219.98	0.0931	9.46%
Fibrinogen, mg/dL	adm	396.0 [330.0–529.5]	390.0 ± 255.83	384.0 ± 146.48	439.0 ± 154.57*	395.0 ± 163.35	393.0 ± 160.1	0.1837	27.32%
	peak	405.0 [331.2–554.0]	395.0 ± 499.63	386.5 ± 168.25	459.5 ± 159.54*	404.0 ± 185.15	445.5 ± 183.97	0.0608	27.32%
**Clinical severity**								**<0.0005**	
Asymptomatic/Mild		343 (61.25%)	160 (65.84%)	96 (64.86%)	44 (45.83%)*	35 (59.32%)	8 (57.14%)		
Moderate/Severe		171 (30.54%)	54 (22.22%)*	45 (30.41%)	43 (44.79%)*	23 (38.98%)	6 (42.86%)		
Critical		46 (8.21%)	29 (11.93%)*	7 (4.73%)	9 (9.38%)	1 (1.69%)	0 (0.0%)		
**Outcome**									
Put on oxygen therapy		112 (20.0%)	50 (20.49%)	27 (18.24%)	24 (25.53%)	8 (13.33%)	3 (21.43%)	0.4331	
Transferred to ICU		72 (12.86%)	38 (15.57%)	12 (8.11%)*	15 (15.96%)	7 (11.67%)		0.1102	
Discharged alive		545 (97.32%)	234 (95.9%)	143 (96.62%)	94 (100.0%)	60 (100.0%)	14 (100.0%)	0.1475	
**Complications**	count	0.0 [0.0-0.0]	0.0 ± 1.71	0.0 ± 0.97	0.0 ± 1.41	0.0 ± 0.64	0.0 ± 0.45	0.3597	
Having any complication		123 (21.96%)	56 (22.95%)	25 (16.89%)	25 (26.6%)	13 (21.67%)	4 (28.57%)	0.4202	
ARDS		76 (13.57%)	39 (15.98%)	13 (8.78%)	17 (18.09%)	7 (11.67%)		0.0873	
Liver dysfunction		54 (9.64%)	25 (10.25%)	9 (6.08%)	16 (17.02%)*	2 (3.33%)	2 (14.29%)	0.0242	

*Statistical data are expressed as IQR, Median ± SD, or absolute number of cases and their percentage in studied sample.

If distribution of variable differs significantly (p < 0.05) in ethnic cohort plotted against all other ones, its value is marked in bold and with asterisk.

Disparities in distribution of data across five ethnic groups are presented with p-values in separate column. p-value is marked in bold if difference between all ethnic groups is statistically significant (p < 0.05).

**Table 4:** Comparison of patients of Middle Eastern subethnicities.

*N*		Total	Arabs	Africans	*p*-value	Missing values
		*n* = 148	114 (77.03%)	34 (22.97%)		
**Patient age**		38.5 [29.0-57.0]	41.5 ± 17.64	34.5 ± 11.69	0.08009	
**Patient gender**	female	93 (62.84%)	71 (62.28%)	22 (64.71%)	0.84238	
	male	55 (37.16%)	43 (37.72%)	12 (35.29%)		
**Comorbidities**						
Current smoking		15 (10.14%)	7 (20.59%)	8 (7.02%)	**0.04529**	
Chronic cardiac disease		10 (6.76%)	1 (2.94%)	9 (7.89%)	0.45502	
Hypertension		37 (25.0%)	2 (5.88%)	35 (30.7%)	**0.00284**	
Asthma		9 (6.08%)	1 (2.94%)	8 (7.02%)		
Chronic kidney disease		4 (2.7%)		4 (3.51%)		
Diabetes		37 (25.0%)	4 (11.76%)	33 (28.95%)	**0.04474**	
Active malignant cancer		3 (2.03%)		3 (2.63%)		
**Physical examination**						
BMI	adm	28.0 [23.62–32.5]	29.0 ± 7.26	27.13 ± 6.33	0.22498	
Body temperature, °C	adm	37.0 [37.0–37.5]	37.0 ± 0.63	37.0 ± 0.62	0.34961	
HR BMP	adm	85.0 [76.0–92.0]	85.0 ± 13.5	85.0 ± 11.65	0.49363	
SBP	adm	120.5 [112.0–131.25]	120.0 ± 16.63	121.0 ± 15.94	0.27736	
SOFA score	adm	0.0 [0.0–0.0]	0.0 ± 0.96	0.0 ± 1.12	0.07010	1.35%
**Laboratory findings**						
WBC, ×10^9^/L	adm	5.2 [4.1–6.4]	5.4 ± 4.15	4.75 ± 2.06	0.12581	0.68%
	min	4.7 [3.6–6.3]	5.1 ± 2.18	4.3 ± 2.27	0.11972	0.68%
Platelets, ×10^9^/L	adm	207.0 [174.0–271.0]	212.0 ± 74.88	202.0 ± 62.49	0.46522	0.68%
	min	211.0 [167.5–263.0]	209.0 ± 79.44	221.0 ± 71.14	0.28758	0.68%
Lymphocytes, ×10^9^/L	adm	1.57 [1.14–2.2]	1.57 ± 0.73	1.64 ± 0.62	0.49267	0.68%
	min	1.5 [0.96–2.1]	1.51 ± 0.7	1.5 ± 0.78	0.31689	0.68%
Neutrophils, ×10^9^/L	adm	2.94 [2.0–3.9]	2.29 ± 1.87	3.11 ± 2.56	0.09368	0.68%
	min	2.57 [1.78–3.7]	2.29 ± 2.06	2.78 ± 1.75	0.07491	0.68%
Neutropenia, <1×10^9^/L	adm	8 (5.41%)	4 (11.76%)	4 (3.51%)	–	0.68%
	min	11 (7.43%)	5 (14.71%)	6 (5.26%)	0.12709	0.68%
NLR	adm	1.89 [1.11–3.09]	1.51 ± 1.93	1.96 ± 2.84	0.20026	0.68%
	min	1.78 [1.11–3.14]	1.5 ± 1.99	1.83 ± 3.14	0.11309	0.68%
LCR	adm	0.25 [0.06–0.97]	0.33 ± 12.11	0.24 ± 17.84	0.19674	8.11%
	peak	15.79 [2.64–57.2]	21.74 ± 52.97	15.26 ± 70.99	0.15690	7.43%
T.bilirubin, μmol/L	adm	8.0 [5.62–10.17]	7.9 ± 5.49	8.05 ± 3.09	0.31502	1.35%
	peak	8.6 [5.93–11.0]	8.95 ± 6.64	8.35 ± 3.53	0.25986	1.35%
ALT, U/L	adm	25.0 [17.0–44.0]	23.5 ± 44.64	32.5 ± 24.03	0.05078	1.35%
	peak	27.0 [19.0–54.0]	25.5 ± 67.1	32.5 ± 10467.88	0.13466	1.35%
AST, U/L	adm	23.0 [18.0–31.0]	23.0 ± 38.5	23.5 ± 16.8	0.09486	1.35%
	peak	25.0 [18.0–36.75]	25.0 ± 45.91	25.5 ± 704.33	0.19697	1.35%
D-dimer, mg/L	adm	0.4 [0.3–0.6]	0.4 ± 0.55	0.3 ± 0.19	**0.01554**	10.81%
	peak	0.4 [0.3–0.7]	0.4 ± 1.34	0.3 ± 3.59	0.07021	10.81%
APTT, s	adm	37.0 [35.0–40.4]	37.3 ± 13.36	37.0 ± 4.84	0.36875	8.78%
	peak	37.6 [35.0–41.3]	37.85 ± 13.83	37.1 ± 9.8	0.37892	8.78%
Creatinine, μmol/L	adm	75.3 [67.0–87.0]	76.0 ± 31.03	75.0 ± 14.8	0.23535	0.68%
	peak	76.0 [67.0–88.0]	77.2 ± 33.37	75.15 ± 21.06	0.25633	0.68%
CK, U/L	adm	84.0 [50.0–140.0]	84.0 ± 255.0	93.0 ± 47.79	0.38742	18.24%
	peak	86.0 [51.25–153.75]	86.0 ± 267.35	100.0 ± 107.07	0.34588	17.57%
CRP, mg/L	adm	5.9 [1.92–19.77]	6.4 ± 52.34	4.0 ± 41.36	0.08961	1.35%
	peak	7.95 [2.12–39.75]	9.05 ± 60.5	5.65 ± 74.93	0.22795	1.35%
LDH, U/L	adm	181.0 [149.0–228.25]	181.0 ± 85.87	166.0 ± 86.73	0.15974	12.16%
	peak	183.0 [149.0–234.75]	186.0 ± 124.13	166.0 ± 1334.94	0.12929	12.16%
Troponin, ng/mL	adm	0.0 [0.0–0.0]	0.0 ± 0.01	0.0 ± 0.0	0.41498	22.3%
	peak	0.0 [0.0–0.0]	0.0 ± 0.06	0.0 ± 0.11	0.36905	22.3%
Ferritin, ng/mL	adm	191.9 [66.0–326.09]	192.31 ± 569.48	158.25 ± 393.37	0.44227	4.73%
	peak	205.0 [77.0–481.0]	205.0 ± 3204.1	194.35 ± 584.34	0.36707	4.73%
Fibrinogen, mg/dL	adm	384.0 [321.75–478.5]	392.0 ± 140.09	326.0 ± 160.17	**0.00758**	24.32%
	peak	386.5 [325.75–510.75]	397.0 ± 156.97	346.0 ± 198.61	**0.01501**	24.32%
**Clinical severity**					0.80646	
Asymptomatic/Mild		119 (80.41%)	28 (82.35%)	91 (79.82%)		
Moderate/Severe		22 (14.86%)	4 (11.76%)	18 (15.79%)		
Critical		7 (4.73%)	2 (5.88%)	5 (4.39%)		
**Outcome**						
Put on oxygen therapy		27 (18.24%)	5 (14.71%)	22 (19.3%)	0.62210	
Patients in ICU		12 (8.11%)	3 (8.82%)	9 (7.89%)	1	
Discharged alive		143 (96.62%)	32 (94.12%)	111 (97.37%)	0.32389	
**Complications**	count	0.0 [0.0–0.0]	0.0 ± 1.88	0.0 ± 1.19	0.37253	
Having any complications		25 (16.89%)	5 (14.71%)	20 (17.54%)	0.79936	
ARDS		13 (8.78%)	3 (8.82%)	10 (8.77%)	1	
Liver dysfunction		9 (6.08%)	3 (8.82%)	6 (5.26%)		

Significant differences between cohorts (p < 0.05) are marked in bold.

*Comorbidities*. Hypertension is the most common comorbidity, present in 20.54% of the study cohort, with no remarkable differences between groups (p = 0.345). Diabetes was present in 17.50% of patients, and its incidence differed significantly among ethnic cohorts (p = 0.0001). The Middle Eastern population had the highest proportion (25%) of patients with diabetes and the prevalence was higher than in the other ethnic groups (p = 0.005). In comparison, East Asians and Caucasians had a substantially lower proportion of patients with diabetes (8.51%, p = 0.01 and 1.67%, p = 0.006 consecutively). Active smoking was present in 6.4% cases and almost half of them were Middle Easterns.

*Symptoms*. Each patient had between two and four symptoms on admission. The most common symptoms were fever (58.04%), followed by cough (53.93%), myalgia (38.93%), sore throat (30%), and shortness of breath (26.96%). The frequency of the symptoms and the values of the body temperature (p = 0.034), pulse (p = 0.001), and respiratory rate (p = 0.039) varied among the ethnic groups. However, the distinction in the major results of the physical examination did not have a clear clinical value. On average, the SOFA score was approximately equal in the ethnic groups on admission (p = 0.273).

*Physical examination*. Middle Eastern patients had a considerably higher average BMI compared to other ethnicities (p = 0.033).

*Laboratory findings*. APTT was longer in East Asians and reached almost the upper limit of the reference range on admission (39.3 ± 4.96 s; p = 0.015). Fibrinogen concentration was also the highest in this ethnic group (p = 0.0045). East Asians had the highest group level of the LDH activity (p = 6.77e 05), which is a non-specific biomarker of a massive tissue breakdown and a predictor of mortality in COVID-19 patients (Yan et al., 2020). Besides this, East Asians had the highest thrombocyte count (p = 4.42e – 06) and a minimal lymphocyte-to-C-reactive protein ratio (LCR) (p = 0.04) both serving as laboratory high-risk complication markers.

On admission, the mean count of leucocytes, neutrophils, lymphocytes, and thrombocytes of the ethnic groups were within the reference range. However, the maximal numbers of WBC and neutrophils were noted in the group of South Asian patients (p < 0.011). Patients of Middle Eastern ethnicity had considerably lower count of thrombocytes (207.0 ± 72.21, p = 0.046), WBC (5.2 ± 3.78, 4.58e – 05), and neutrophils on admission (2.94 ± 2.43, p = 1.88e – 05). In this group, the percentage of people with moderate and severe neutropenia (<1.0×10^9^/L) was distinctly higher than in the other groups (5.41%, p = 0.008). This accounted for the lowest neutrophil‐to‐lymphocyte ratio (NLR) in the Middle Eastern patients (1.89 ± 2.66, p = 0.001). The tendency remained the same at the peak of the disease.

*Disease severity*. Almost two-thirds of our cohort (61.25%) were asymptomatic or had mild symptoms. Moderate-to-severe disease was seen in 30.54% of the cohort, and 8.21% were critical. There was a marked disparity in the distribution of patients from distinct severity levels in the ethnic groups (p < 0.0005). In Caucasians, the portion of patients diagnosed with moderate-to-severe disease was higher than in South Asians and Middle Easterns (38.98% vs. 22.22% and 30.41%) despite the least number of comorbidities in the Caucasian group. This was also noticeable in East Asian patients. On the contrary, patients from the Middle East had a higher number of comorbidities (chronic cardiac disease 7%, diabetes 25%, smoking 10%), and essentially higher mean BMI (see above), yet they had a much lower proportion of patients with critical disease course—4.73% vs. 11.93% in South Asians, and 9.38% in East Asians.

*Disease outcome*. There was no marked difference between ethnic groups in primary outcome of COVID-19 (p = 0.147). The overall mortality was 2.68%. Twenty percent of the total cohort required oxygen supplementation and a lower proportion of patients from the Middle East required ICU admission compared to the other groups (8.11% vs. 12.86% in the overall sample; p = 0.044). The rate of complications was similar in different ethnic groups except for liver dysfunction which was observed in a higher proportion of East Asian patients (17.02% vs. 9.64% in the total cohort; p = 0.008).

### 4.2 Ranking the Most Important Features

**Figure 4:**
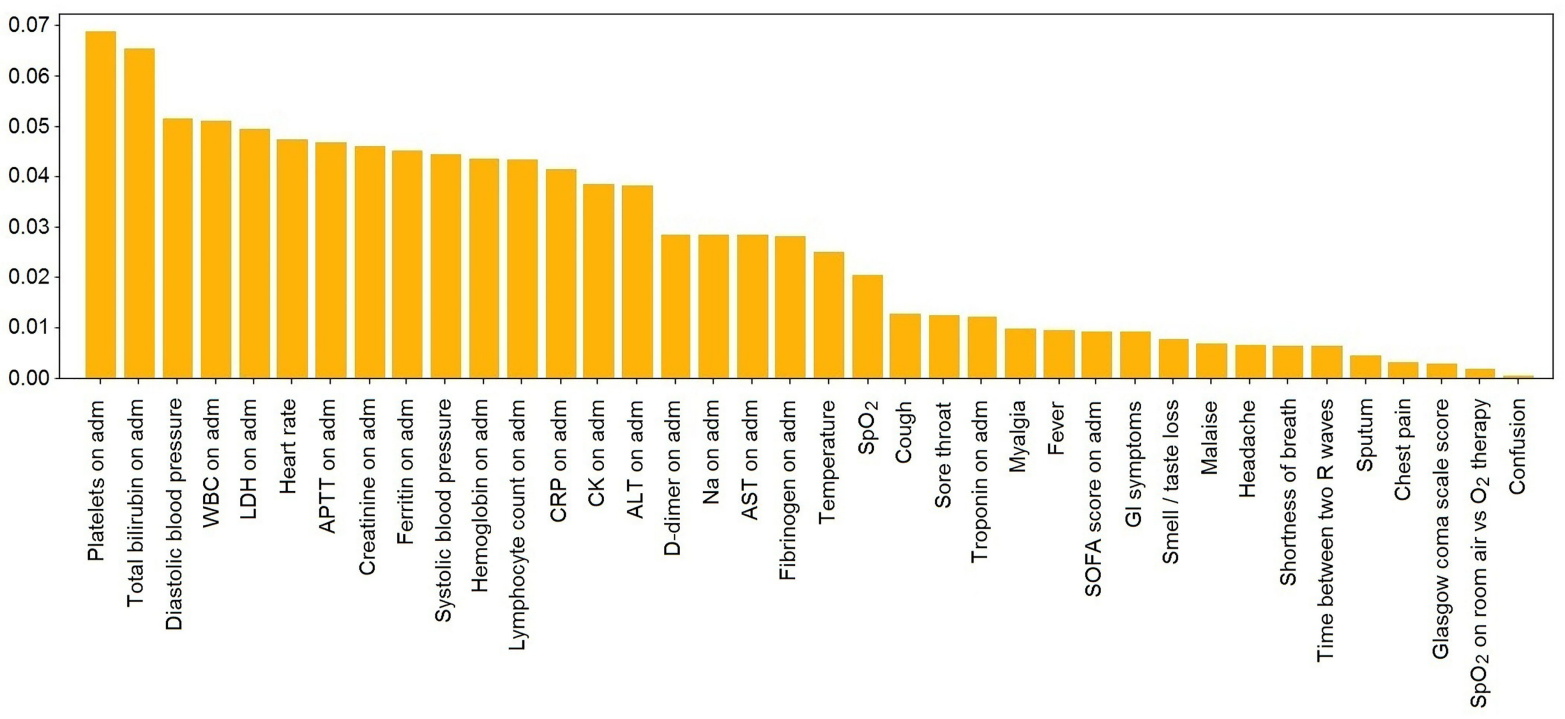
Performance of employed NN classification method.

**Table 5** and **Figure 4** display the values of impurity-based attribute ranked averaged across four tree-based ML classifiers (Random Forest, AdaBoost, Gradient Boosting, and ExtraTrees).

**Table 5:** Ranking scores of variables selected for ML model.

Score	Feature	Score	Feature	Score	Feature	Score	Feature
0.06883	Platelets	0.04361	Hemoglobin	0.02051	SpO_2_	0.00644	Headache
0.06522	Total bilirubin	0.04336	Lymphocytes	0.01256	Cough	0.00634	SOB
0.05150	DBP	0.04148	CRP	0.01248	Sore throat	0.00634	RR/min
0.05104	WBC	0.03849	CK	0.01215	Troponin	0.00446	Sputum
0.04947	LDH	0.03812	ALT	0.00988	Myalgia	0.00300	Chest pain
0.04722	HR BPM	0.02838	D-dimer	0.00942	Fever	0.00287	GCS
0.04681	APTT	0.02825	Na	0.00931	SOFA score	0.00187	SpO_2_ on RA vs.
0.04594	Creatinine	0.02825	AST	0.00925	GI symptoms		O_2_ therapy
0.04503	Ferritin	0.02822	Fibrinogen	0.00761	Smell/taste loss	0.00046	Confusion
0.04428	SBP	0.02483	Temperature	0.00672	Malaise		

### 4.3 Classification Concerning Ethnicity With Neural Network

To evaluate the classifier output quality, we trained several ML classification models using a stratified 10-fold cross-validation technique to generalize the models to the true rate error. For each fold, we used 90% of the data to train the model and then tested it on the remaining 10%. The decision matrices built on the test dataset for all folds were combined and used to calculate the performance metrics. The best performance measures were obtained with a three-layer fully connected neural network (NN).

**Figure 5:**
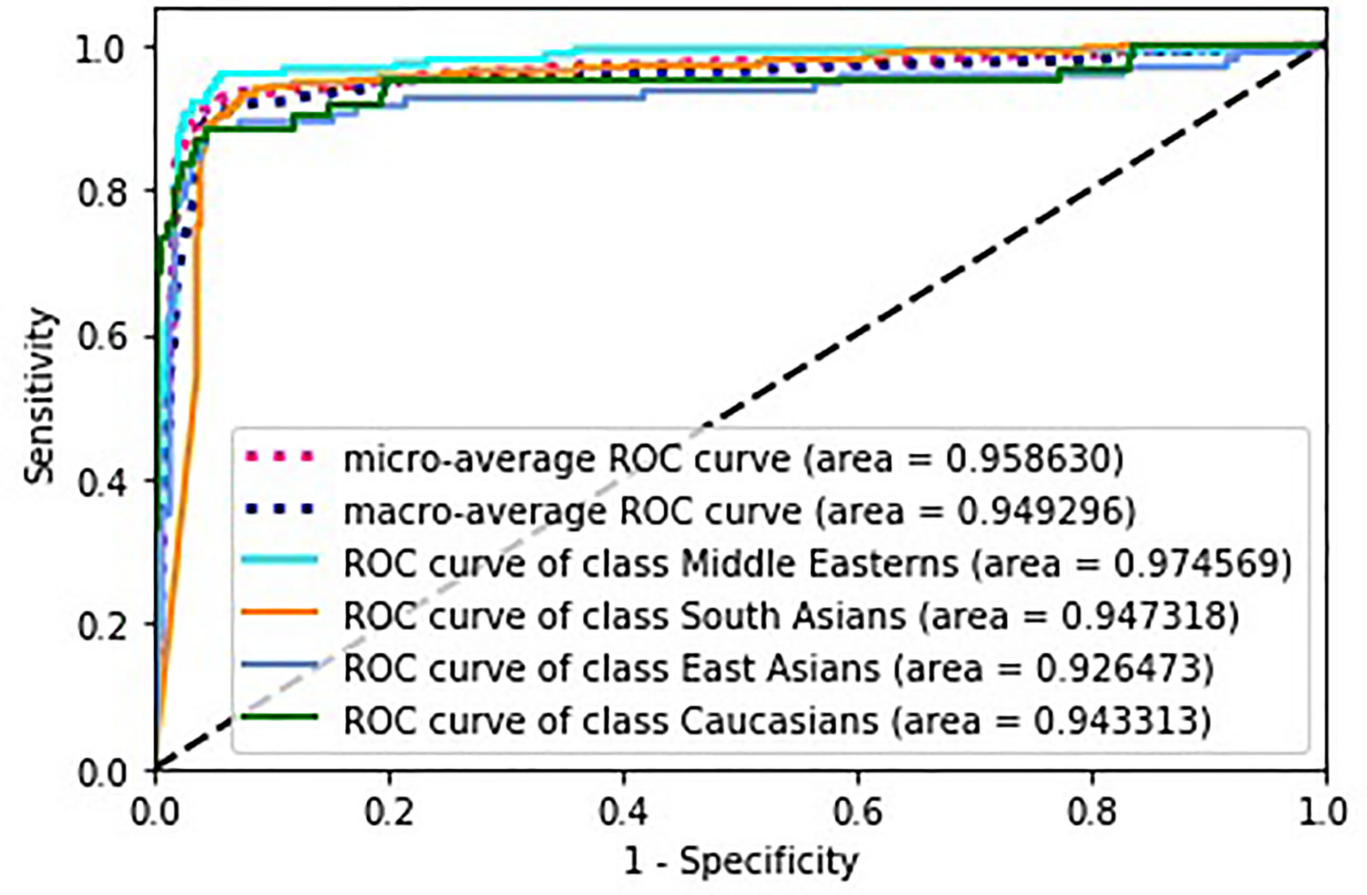
Performance of neural network classification model.

**Figure 5** depicts receiver operating characteristics (ROC) for multi-class classification model. To generalize the area under the ROC curve (AUC) for the multi-classification problem, the average AUC of all possible pairwise combinations of groups was computed, and then unweighted mean was considered as a metric. In the figure, we also present micro-average (aggregates the contributions of all classes to calculate the metric) and macro-average (computes the metric independently for each class and then takes the average) AUCs.

**Table 6** lists the confusion matrix of the trained model for each group, indicating true-positive, true-negative, false-positive, and false-negative numbers. Each row of the error matrix represents the actual class, while each column shows the instances in a predicted class. Precision, recall, harmonic score, accuracy, macro average (unweighted mean per class), and weighted average (support-weighted mean per group) of the classification performance are specified in **Table 7**.

**Table 6:** Confusion matrix to assess accuracy of prediction with three-layer dense NN model classifying cases by ethnic group.

		Predicted			
		Middle Easterns	South Asians	East Asians	Caucasians
**Actual**	Middle Easterns	121	22	2	3
	South Asians	4	232	5	3
	East Asians	5	6	82	1
	Caucasians	2	3	1	54

**Table 7:** Classification metrics of neural network model.

	Precision	Recall	F1 score	Support
South Asians	0.88	0.95	0.92	244
Middle Easterns	0.92	0.82	0.86	148
East Asians	0.91	0.87	0.89	94
Caucasians	0.89	0.90	0.89	60
Accuracy			0.90	546
Macro average	0.90	0.89	0.89	546
Weighted average	0.90	0.90	0.89	546

## 5 Discussion

### 5.1 The Comparison of the Ethnic Groups

An ethnic group is a group of people whose members identify with one another through common cultural heritage. This term usually reflects a shared culture and social behavior; however, it can also be used to imply variations in genetic makeup between different groups. Several studies showed remarkable ethnicity-related differences in clinical features of various diseases. For example, deaths for Hepatitis C were higher among Native Americans and Blacks compared to Caucasians. Studies demonstrated that an immunologic basis can explain this difference (Sugimoto et al., 2003). Moreover, during the 2009/2010 influenza pandemic, differences in mortality rates were observed, where non-Caucasians had a markedly higher mortality rate compared to Caucasians (Zhao et al., 2015).

#### 5.1.1 Genetic Factors

Genetic factors may account for ethnic disparities. However, the variation in genes within ethnic groups can also be high, especially in Arabs, unified by the Arabic language rather than a common origin. The ethnic group primarily inhabits 22 member states of the Arab League in Western Asia and North Africa (Frishkopf, 2010). Though the majority of the North Africans speak Arabic as their native language, they have a Berber (not Arab) origin. Reasonably, there are genetic disparities among Arab nationalities despite cultural, geographic, and linguistic similarities among them. To study Arabian genealogy, geneticists analyze Y-DNA haplogroup tree (Mahal and Matsoukas, 2018). With this method, they showed that the haplotype of Jordanian bedouins had its traces in Palestinians, Yemeni, Moroccan, Libyans, and Tunisians. There was a low genetic diversity among these subethnicities (Almahasneh et al., 2018). Another study justified genealogical relatedness between Iraqi and Kuwaiti individuals. A non-significant genetic distance was shown for the following ethnic pairs: Northern Iraqi and Lebanese, Kurdish and Iranian, Iraqi and Iranian (Dogan et al., 2017).

Analogously, the genetic background of the UAE citizens was influenced by the neighboring countries and remote geographic regions. In males, Y haplogroups had similarities with the Middle Eastern, Central, and South Asian genes. Fifty-two percent of Emirati men had the Middle Eastern haplogroup J, 21% of them inherited the E haplogroup common in West and East Africa, and 14% of the individuals had the R haplogroup originated from Central and South Asia and Eastern Europe (Daw Elbait et al., 2021). A close genetic distance between inhabitants of distinct Arab countries denoted that they had a common genetic background. This enabled us to analyze data for Arabs without dividing them into subethnicities in the current study. To identify a genetic background of each individual accurately, we would require expensive genetic testing.

There has been an abundance of publications on COVID-19; however, data on ethnicity and COVID-19 remain limited. Observations from the UK and the USA highlighted increased disease severity and mortality among Blacks, Asians, and Minority Ethnic groups (BAME) (Pan et al., 2020; Lab, 2020). Some authors analyzed peer-reviewed literature to study the effect of ethnicity on COVID-19 outcomes and found no differences (Pan et al., 2020). The same scientific group inspected preprint articles, some of which suggested poorer outcomes in BAME compared to White patients. These publications compared Caucasians to non-Caucasians, mostly Asians, Blacks, and Hispanics.

No studies ascertained specifically the morbidity and mortality of Middle Eastern patients during the COVID-19 pandemic. We believe our findings are of particular interest as this ethnic group displays a higher risk factor profile, yet fewer patients had a critical disease course compared to other groups. We also identified differences between South Asian and East Asian ethnic groups. These two distinct ethnic groups are often considered as one common group in most publications.

Literature on systemic hypertension and cardiovascular disease reflected considerable variation in disease manifestation, outcome, and response to different pharmacological agents among different ethnic groups. A relevant example is that ACE-I and ARB medications were less effective in reducing blood pressure in patients of African ethnicity. In fact, these patients had worse cardiovascular outcomes when started on ARB monotherapy (Brewster et al., 2016).

Angiotensin-converting enzyme 2 (ACE2) receptor is thought to play a critical role in the pathogenesis of COVID-19 as SARS-CoV-2 uses the ACE2 receptor for cell entry (Hoffmann et al., 2020). The virus also uses transmembrane serine protease (TMPRSS2) and Furin peptidase to invade human cells (Al-Mulla et al., 2021). Different studies finished up with inconsistent findings on the genetic predisposal and protection against SARS-CoV-2 among ethnic group in multinational countries. For example, in the USA, African Americans at a higher risk of COVID-19 compared to other ethnicities that live in the country (Whites, Asians, American Indians, Alaskan Natives, and other minorities). This might be explained by the increased gene expression of ACE2 and TMPRSS2 genes in this ethnic group. Additionally, African Americans with asthma are at a greater risk of suffering from the severe form of COVID-19 (Peters et al., 2020). African Americans and Whites have a lower ACE2 expression cell ratio than Asians (Cao et al., 2020). The distribution of ACE1 and ACE2 genotype rates matches CFR in various countries (Gupta and Misra, 2020). The highest CFR (9.6%) is in Europe, followed by North America (5.9%) and Asia (3.5%) (Dongarwar and Salihu, 2020). In a multi-ethnic society, the highest CFR is registered in Blacks (Goldstein and Atherwood, 2020). This suggests gene–environment interactions and ethnic disparities in immune response to COVID-19 (Goldstein and Atherwood, 2020; Nepomuceno et al., 2020).

Factors that influence ACE2 receptor expression, such as the use of ACE-I and ARB, are supposed to affect disease course and severity (Madjid et al., 2020). Gene variation can explain variance of susceptibility to SARS-CoV-2 among ethnicities (Cao et al., 2020).

A thought-provoking question that presents itself is whether patients from the Middle East have an ACE2 receptor morphology that is protective against developing a more severe COVID-19 disease. Researchers investigated whole-exome sequences of individuals from Middle Eastern populations to explore natural variations in the ACE2. They identified two activating variants in the ACE2 gene: K26R and N720D. The variants are more common in Europeans and rare in the Middle Eastern, East Asian, and African populations. The variants change ACE2 gene expression and make people more vulnerable to SARS-CoV-2 infection. Previous studies suggest that K26R can activate ACE2 and facilitate binding to the receptor binding domain while N720D enhances TMPRSS2 protease cutting (Al-Mulla et al., 2021). K26R variant occurs in European people with a frequency of about 0.5%, which predisposes them to more severe SARS-CoV-2 disease. Another single-nucleotide polymorphism of ACE2 that may genetically protect from SARS-CoV-2 disease is more common in African people with a frequency of about 0.3% (Calcagnile et al., 2020). In contrast, deleterious variants that suggest a possible decrease in Furin protease function are detected more frequently among Middle Easterns than Europeans (Al-Mulla et al., 2021).

#### 5.1.2 Socioeconomic Factors

Disparities in SES can also account for ethnic and race disparities in COVID-19. Previous research highlighted a strong association between SES and disease outcomes. The ethnic groups with the lower SES are at risk of contracting COVID-19 (Garg, 2020; Kopel et al., 2020). It remains unclear whether this can be explained by a host genetic interaction (e.g., higher prevalence of underlying chronic disease) or non-genetic behavioral factors such as higher-density living, the use of public transportation, and possibly lower health literacy (Singu et al., 2020). Data related to SES in the UAE (e.g., the level of education and the monthly income) are not routinely collected in hospital medical records so it remains indistinguishable whether SES affected disease severity in our study or not. However, this seems unlikely as the South Asian patients (who represent 43% of our cohort) were younger and had no considerable comorbidities, but had a similar disease outcome to other ethnic groups.

The UAE is a high-income country that has a high rate of young people and a disproportion between men and women due to the recruitment of male workforce (Paulo et al., 2017; Paulo et al., 2018). Such distribution of males and females can explain the prevalence of men admitted to the Mediclinic Parkview hospital, which was used as a research center for our study. Apart from gender and age disparities, the UAE has an uneven distribution of residing nationalities. Emirati citizens make up 11.48% of the population whereas most residents come from India (27.49%) and Pakistan (12.69%), and Egyptians constitute the largest diaspora among non-Emirati Arabs (4.23%) (UAE Population Statistics, 2021).

Although health insurance is mandatory, there is a wide range of insurance providers, and continuous care of expatriates is not well maintained (Paulo et al., 2017; Paulo et al., 2018). With a new place of affiliation, an employee gets a new insurance plan (How to Get Health Insurance in the UAE? News, 2021) which depends on a job role and official monthly income associated with it. The lower the job grade is, the narrower is the insurance coverage. To improve the situation, some companies unified insurance plan for all their employees.

#### 5.1.3 Hematological Abnormalities

COVID-19 can manifest with a profound inflammatory response, which may cause severe immune damage to the lungs. Coronaviruses are able to infect bone marrow cells, which can result in abnormal hematopoiesis (Desai et al., 2021). That is why SARS-CoV2 infection can cause several hematological abnormalities (Mank et al., 2021). The most common abnormalities in COVID-19 include neutrophilia, lymphopenia, and thrombocytopenia. WBC count can be normal or decreased upon admission, and it increases with disease progression. Also, an elevation in the WBC count can be caused by co-infections or medications (e.g., prednisone) (Khartabil et al., 2020).

*Lymphopenia* leads to the dysfunction of immune system in severe COVID-19 and makes the patients vulnerable to bacterial infections (Chen et al., 2020; Sun et al., 2020). Pronounced lymphopenia and thrombocytopenia carry poor prognosis especially if accompanied by the elevated D-dimer level (Desai et al., 2021). Both neutrophilia and neutropenia are predictive of poor outcomes and severe respiratory failure in this category of patients (López-Pereira et al., 2020). However, neutropenia is less common in COVID-19. There were only a few reports of the decreased neutrophil count in these patients (Ai et al., 2020; Ahnach et al., 2020; Yarali et al., 2020). The exact reason for neutropenia in the disease remains unknown. The suggested mechanisms of neutropenia development include bone marrow suppression and accelerated peripheral destruction of neutrophils. These mechanisms have been well described in other viral infections including HIV, cytomegalovirus, Epstein–Barr virus, viral hepatitis, and influenza (Munshi and Montgomery, 2000). Both the moderate (<1,000 cells/μl) and especially the severe neutropenia, which is also called agranulocytosis (<500 cells/μl), are conditions with an extraordinary risk of infections. The conditions require patient monitoring and empirical antibiotic therapy along with the administration of granulocyte colony-stimulating factor in some cases (Devi et al., 2021).

The neutrophil‐to‐lymphocyte and lymphocyte-to-C-reactive protein ratios are well-established inflammation markers that reflect systemic inflammatory response (Lagunas-Rangel, 2020). NLR is a widely used biomarker for assessing the severity of bacterial infections (Naess et al., 2017; Sun et al., 2020). The increase in neutrophil count indicates the disease aggravation. The decrease in lymphocyte count denotes impairment in immune functioning (Celikbilek et al., 2013; Huang et al., 2019). NLR is shown to be an independent risk factor of severe COVID-19 (Borges et al., 2020). The ratio increases dramatically in patients with the severe disease form (Lagunas-Rangel, 2020). The lymphocytopenia and the increase in the NLR are the most obvious hematological abnormalities associated with the disease.

The low LCR along with the high NLR suggest a poor prognosis in COVID-19 patients (Lagunas-Rangel, 2020). The LCR can capture the early part of the inflammatory cascade more sensitively than the NLR as the CRP levels have been shown to rise earlier than either neutrophilia or lymphopenia is seen in the course of disease. The low LCR and the high NLR observed at different time frames can be regarded as independent predictive markers for in-hospital complications and mortality in COVID-19 patients (Liu et al., 2020).

In our study, the minimal neutrophil count and the maximal percentage of cases with neutropenia (<1.0×10^9^/L) were observed in the group of Middle Eastern patients. Among 10 patients with neutropenia, 2 presented with the severe disease and died, 7 patients had comorbidities, and 3 of them developed complications. The NLR was also minimal in the Middle Eastern group. A rise in NLR across the disease as well as high initial levels of the NLR are the markers of poor disease outcomes and high mortality. This finding is aligned with the fact that the Middle Eastern group had the lowest number of patients who required intensive care and developed the critical disease.

In the group of East Asians, we observed the minimal values of LCR and the tendency toward the highest NLR. This correlates with the high proportion of patients with the moderate and severe disease and the maximal number of patients who developed liver dysfunction in this ethnic group.

Both LCR and NLR should be interpreted in conjunction with the clinical data to identify patients at risk of poor prognosis of COVID-19. Neutropenic conditions should be followed up to prevent concomitant infections worsening the disease severity.

### 5.2 The Top-Ranked Features of the Model for Classification Concerning Ethnicity

The top-ranked features listed in **Table 5** may represent the ethnic-specific response to the disease. Notably, the count of platelets was the top ranked variable in the model that reflects ethnic-specific features of COVID-19. Because of the disturbed coagulation in COVID-19 patients, there are considerations for the potential role of platelet function and/or platelet activation in the disease severity (Larsen et al., 2020). Furthermore, APTT is also a valuable feature of the classification model (the 7th one out of a total number of 38).

The WBC count and the level of lymphocytes on admission are also among the top-ranked attributes. The facts support the hypothesis that some mechanisms of the immune response to COVID-19 are specific to the ethnicity of the patient. Meanwhile, lymphopenia is known to be an essential clinical feature in patients with severe SARS-CoV-2 infection (Zheng et al., 2020).

The activity of LDH enzyme ranks 5th among the most valuable predictors. The biochemical constants (e.g., total bilirubin and creatinine concentration) may account for genetic-based differences in the enzyme regulations and metabolism. The presented symptoms, SOFA, and GCS scores are at the bottom of the list of the valuable features; i.e., the clinical appearance of COVID-19 is not specific to the patient’s ethnicity.

### 5.3 The Classification Model and Its Performance

To check the quality of the outcome of the supervised ML model, we employed several algorithms and compared their performance. The NN outperformed all the other methods. We tuned parameters of the model in terms of the number of hidden layers and neurons, optimizer, and hyperparameters and built the three-layer fully connected NN. It showed up to 90% averaged accuracy in the classification by the ethnic group.

The high accuracy of the model supports our hypothesis of the occurrence of ethnic-specific features and patterns in the dataset. As seen from the error matrix (**Table 6**) and performance matrix (**Table 7**), the best performance is shown for the most numerous class of South Asians. The highest rate of false-positive values was obtained for the Middle Eastern class, which comes second in terms of the number of patients. The misclassification can be explained by some similarities between the two classes rather than overfitting of the ML algorithm.

To assess the performance of the model, we built the ROC curves for each class separately and calculated the appropriate AUCs for micro and macro average. **Figure 5** clearly indicates the high performance of the model with regard to an ethnic group. Micro averaged curve and its AUC indicate high performance for each group as it is calculated globally.

## 6 Conclusion

- In our cohort, Caucasian or East-Asian COVID-19 patients tended to have a more severe disease despite a lower risk profile. In contrast to this, Middle Eastern COVID-19 patients have a higher risk factor profile but they did not differ markedly in disease severity from the other ethnic groups.
- The accurate ethnicity classification model, which is based on the laboratory, physical, and clinical findings, reveals the presence of ethnic-specific features of COVID-19.
- The high performance of the ML NN method applied to the classification by the ethnic group from the laboratory and clinical findings supports the occurrence of features and patterns that are specific to ethnicity. This may impact the development of medical treatment and protocols based on ethnic background.
- Larger studies are needed to explore the role of ethnicity in COVID-19 disease features.

## 7 Limitations

One of the major strengths of the study was the recruitment of a cohort reflective of all adult age groups. This enabled us to calculate actual risk estimates. The *second* positive is that all the patients diagnosed with COVID-19 were hospitalized regardless of their disease severity. The diagnostics was performed in full accordance with the common “National Guidelines for Clinical Management and Treatment of COVID-19” (National Emergency Crisis and Disasters Management Authority, 2020), which provided us with the unique study cohort representative of the adult population.

The current study has several limitations. *First*, it is a single-center study in the Emirate of Dubai, which is the most populated city in the UAE with the highest percentage of expatriates (91%), and it does not cover other cities such as Ras-Al-Khaimah where expatriates make up 69% of the population. Thus, UAE nationals might be underrepresented in this cohort.

*Second*, we were unable to assess the possible impact of socioeconomic factors. The relationship between ethnic background and socioeconomic status with health outcome is complex and multidimensional. Data on socioeconomic status in the UAE (e.g., the level of education and the monthly income) are not routinely collected in hospital medical records. Although we consider its influence on the health state of people with different ethnicity, it is impossible to estimate the effect of the aspect within the society of Dubai. The information on personal income does not reflect the spectrum of expenditures by an individual. Thus, the above-mentioned factors should be the focus of a separate study on economics and public health. Information on the socioeconomic status is missing in the dataset analyzed. The health insurance plan is not a valid marker of socioeconomic status in the UAE (see the *Discussion* section) and the UAE government provided free medical care to all COVID patients during the study period.

*Third*, there is no reliable and affordable tool for segregating examinees into ethnic groups and subethnicities. Apart from geographic and cultural similarities, ethnicities have common genes that were not analyzed in the current study. Since the data on the patients’ Y haplogroups were not available, we divided the study cohort by geographic location. Large ethnic groups were used in this study. This allowed us to build accurate classification models that justified an association between the disease course and ethnicity. However, we were unable to analyze statistics on COVID-19 in distinct nationalities as the correspondent subgroups were low in numbers and unbalanced.

*Fourth*, although scientists pay much attention to the association of genetic (e.g., ACE2) factors with the COVID-19 severity and outcomes, the settings of our study did not allow us to focus on this aspect. Genetic tests are quite expensive procedures and are not covered by health insurance. During the first wave of the COVID-19 outbreak, genetic factors were not the focus of research activities. As the pandemic evolves, the analysis of such factors may be helpful for the healthcare sector in multinational countries including the UAE.

## Data Availability Statement

The datasets generated for this study are available on request at the site of Big Data Analytics Center (https://bi-dac.com/covid19-dubai-dataset/).

## Ethics Statement

The studies involving human participants were reviewed and approved by Mediclinic Middle East Research and Ethics Committee (MCME REC) (reference number MCME.CR.104.MPAR.2020), Dubai Scientific Research Ethics Committee (DSREC), and Dubai Health Authority (protocol number DSREC-05/2020_25). Written informed consent for participation was not required for this study using secondary deidentified data in accordance with the national legislation and the institutional requirements.

## Author Contributions

ML, NS, RS, DK, and SN collected the dataset. FA and YS wrote the manuscript. TH performed the statistical analysis, prepared the figures and tables for data presentation and illustration. TT analyzed the hematological findings and contributed to writing Results and Discussion sections. NZ, TL, and DS contributed to the literature review and data analysis. All authors contributed to the article and approved the submitted version.

## Conflict of Interest

The authors declare that the research was conducted in the absence of any commercial or financial relationships that could be construed as a potential conflict of interest.

## Publisher’s Note

All claims expressed in this article are solely those of the authors and do not necessarily represent those of their affiliated organizations, or those of the publisher, the editors and the reviewers. Any product that may be evaluated in this article, or claim that may be made by its manufacturer, is not guaranteed or endorsed by the publisher.

## Abbreviations

ACE, angiotensin-converting enzyme; ACE-I, angiotensin-converting-enzyme inhibitors; Adm, admission to the hospital; ALT, alanine aminotransferase; APTT, activated partial thromboplastin time; ARB, angiotensin-II receptor blockers; ARDS, acute respiratory distress syndrome; AUC, area under the ROC curve; AST, aspartate aminotransferase; BAME, Blacks, Asians, and Minority Ethnic groups; BMI, body mass index; CFR, case fatality ratio; CK, creatine kinase; COVID-19, coronavirus disease 2019; CRP, C-reactive protein; DBP, diastolic blood pressure; GCS, Glasgow coma scale; HR BPM, heart rate beats per minute; ICU, intensive care unit; IQR, interquartile range; LDH, lactate dehydrogenase; LCR, lymphocyte-to-C-reactive protein ratio; ML, machine learning; NLR, neutrophil‐to‐lymphocyte ratio; NN, neural network; PCR, reverse transcription-polymerase chain reaction; ROC, receiver operating characteristic; RR/min, the time elapsed between two successive R-waves on the electrocardiogram; SARS-CoV-2, severe acute respiratory syndrome-related coronavirus 2; SBP, systolic blood pressure; SD, standard deviation; SES, socioeconomic status; SOB, shortness of breath; SOFA, Sequential organ failure assessment; SpO_2_, oxygen saturation; TMPRSS2, transmembrane serine protease 2; WBC, white blood cells.
